# Geometric Facial Gender Scoring: Objectivity of Perception

**DOI:** 10.1371/journal.pone.0099483

**Published:** 2014-06-12

**Authors:** Syed Zulqarnain Gilani, Kathleen Rooney, Faisal Shafait, Mark Walters, Ajmal Mian

**Affiliations:** 1 School of Computer Science and Software Engineering, The University of Western Australia, Perth, Western Australia; 2 School of Anatomy, Physiology and Human Biology, The University of Western Australia, Perth, Western Australia; 3 Cranio-MaxilloFacial Unit, Princess Margaret Hospital for Children, Perth, Western Australia; IIT - Italian Institute of Technology, Italy

## Abstract

Gender score is the cognitive judgement of the degree of masculinity or femininity of a face which is considered to be a continuum. Gender scores have long been used in psychological studies to understand the complex psychosocial relationships between people. Perceptual scores for gender and attractiveness have been employed for quality assessment and planning of cosmetic facial surgery. Various neurological disorders have been linked to the facial structure in general and the facial gender perception in particular. While, subjective gender scoring by human raters has been a tool of choice for psychological studies for many years, the process is both time and resource consuming. In this study, we investigate the geometric features used by the human cognitive system in perceiving the degree of masculinity/femininity of a 3D face. We then propose a mathematical model that can mimic the human gender perception. For our experiments, we obtained 3D face scans of 64 subjects using the 3dMDface scanner. The textureless 3D face scans of the subjects were then observed in different poses and assigned a gender score by 75 raters of a similar background. Our results suggest that the human cognitive system employs a combination of Euclidean and geodesic distances between biologically significant landmarks of the face for gender scoring. We propose a mathematical model that is able to automatically assign an objective gender score to a 3D face with a correlation of up to 0.895 with the human subjective scores.

## Introduction

Cognitive judgements of facial attractiveness, gender and the degree of masculinity/femininity are found to be universally reproducible in people of varied cultural and ethnic backgrounds [1], [2]. The Human mind has the capability to assess facial masculinity/femininity and this gender attribute plays an important role in social behaviours. Psychologists and cognitive scientists have extensively analysed the role of perceived gender (masculinity/femininity) on various socio-psychological behaviours in a number of studies (see Table. 1 for a summary).

**Figure 1:**
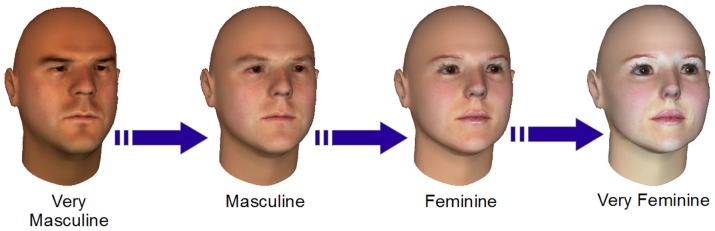
Facial gender is considered to be a continuum over masculinity or femininity. Figure shows morphed 3D images of the same individual with gender varying from highly masculine to highly feminine. Which geometric features do human observers employ for ascribing a score to this variation and can such scores be replicated by computer algorithms? (Note: These images have been created from a model [45], [46] as we are barred from publishing images of actual subjects under ethics approval.)

A subjective gender score is a tangible metric that human raters assign to the degree of masculinity/femininity of a face. This is because, though sex is binary, gender is understood to be a continuum. For example, Figure 1 shows synthetic images of the same individual by varying its gender from very male to very female. In the literature these scores have also been referred to as perceptual gender scores, masculinity/femininity scores or masculinity/femininity index (referred later as masculinity index for brevity).

Subjective gender scoring has been widely used by researchers in Psychology to study the relationship between sexual dimorphism and facial attractiveness [3], [4], mate choice [5], [8], personal character traits [9] as well as perceived and actual health [10]. Applications of subjective gender scores in medical and health care include analysis of the effects of syndromes (e.g. Autism Spectrum Disorder) on facial masculinity/femininity [11], relationship between sexual dimorphism and semen quality [12]/facial symmetry [13]. Other uses include evaluation of the outcome of facial cosmetic surgery [14], [15]. A comprehensive overview of the applications of subjective gender scoring is given in Table 1. In these studies, a number of human raters are asked to judge the masculinity/femininity of the subjects.

**Table 1:** Application of masculinity/femininity ratings in various fields of research.

Study	Reference	Subjects	Raters	Ratings
Correlation between masculinity and trustworthiness/emotions	[9]	12	40	480
Relationship between masculinity/femininity and attractiveness as well as masculinity and distinctiveness	[3] [4]	71	204	5036
Relationship between masculinity/femininity and health	[10]	310	37	11470
Relationship between masculinity/femininity and symmetry.	[13]	194	39	5599
Role of gender scores in sex classification of faces.	[47]	200	40	8000
Relationship between sexual behaviour and masculinity/femininity	[5]	362	109	40952
Womens' preference and mate choice based on masculinity of men	[48] [7]	40	20	800
Relationship between masculinity and semen quality in men	[12]	118	12	1416
Relationship between sociosexuality and gender ratings	[6]	8+50	195+17	2410
Role of masculinity in the functioning of a male endocrine system	[19]	57	72	4104
Role of masculinity and femininity in distinguishing homosexuals	[49]	95	58	5510
Effects of syndrome on masculinity/femininity	[11]	103	8	824
Comparison between masculinity (attractiveness) and intelligence as cues for health and provision of resources in mate selection	[8]	32	689	22048
Evaluating the outcome of facial cosmetic surgery in terms of perceptual attractiveness; pre and post surgery	[15] [14]	32; 20	163; 90	5216; 1800

Applications of perceptual gender ratings by employing human raters. Notice the huge number of ratings performed in case. References are provided for interested readers.

The process of perceptual gender rating in itself is both time and resource consuming and a challenging problem is to identify the nature of predictors or features that are employed by the human mind for this task. Some researchers have also investigated objective scores for sexual dimorphism (masculinity/femininity) using morphometric analysis [16]–[18]. The key idea behind calculating objective masculinity index is to use facial measurements, like distances between biologically significant landmarks or ratios of these distances, for obtaining a score of facial masculinity/femininity. For each face these measures can be used individually or collectively by adding their standardised measures or their Z-scores.

Scheib et al. [16] obtained masculinity indices by summing up the standardized facial measures of the cheek-bone prominence and relative lower face length from grayscale pictures of 40 male subjects. The authors then asked 12 female participants to rate these faces for attractiveness. Interestingly, the masculinity index correlated positively with facial attractiveness (more masculine males were more attractive) which is against the established norms [3]. In a similar study, Penton et al. [17] calculated five separate masculinity indices for each face using measures related to eye size, ratio of lower face height to total face height, cheek bone prominence, ratio of face width to lower face height and mean eyebrow height. Two dimensional pictures of 60 male and 49 female faces were used in this study. The authors did not find a correlation between these five dimorphic measurements and female-rated facial attractiveness. However, the rated attractiveness correlated positively with a composite masculinity index found by summing up the standardized Z-scores of the five individual measures. In a later study, Pound et al. [19] used the same approach to calculate a composite facial masculinity index from 2D photographs of 57 male subjects. The study aimed at analysing the correlation between circulating testosterone levels and masculinity in males. Fifty seven male subjects were first asked to predict, by seeing the photographs, the outcome of a particular wrestler in six wrestling bouts. Subjects were then shown videos of the bouts allocated to a “winning” and “loosing” condition and pre/post task testosterone levels were measured. A group of 72 participants was then asked to rate the subjects for their perceived masculinity. The authors did not find any correlation between perceived masculinity and pre/post task testosterone levels. However, post task increase in testosterone levels correlated positively with the facial masculinity index. Note that, none of these studies explored the relationship between the perceived/rated masculinity and the objective facial masculinity index.

A more sophisticated method of obtaining the masculinity index is to first perform sex classification using discriminant analysis and then use the discriminant scores associated with each face as its masculinity index. One of the earlier attempts in that direction was made by Burton et al. [20]. The authors performed sex classification on 179 faces using a set of 16 2D and 3D Euclidean facial distances as well as their ratios and angles. The discriminant function score of each face was taken as its masculinity index and the reported sex classification accuracy using Discriminant Function Analysis (DFA) was 94%. However, the authors could not find a positive correlation between their objective scores and the perceptual subjective scores obtained by asking 13 participants to rate the subjects' faces for masculinity/femininity. The correlation coefficient was 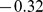 for male faces and 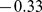 for female faces. In another study, Thornhill and Gangestad [18] used DFA based on five measures of masculinity (chin length, jaw width, lip width, eye width, and eye height) to yield 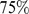 sex classification accuracy on 2D images of 295 subjects. Discriminant function scores were then used to measure facial masculinity. The authors then analysed the relationship between these masculinity scores and health in terms of respiratory diseases and their duration. There was a significant negative correlation for men and positive for women, between health and facial masculinity. Note that Rhodes et al. [10] did not find any such correlation between perceived masculinity and the actual health of female subjects.

A similar technique was employed by Scott et al. [21] to obtain a morphometric masculinity index. Two datasets of textured images of 20 male faces and 150 (75 male/75 female) faces were used for this purpose. Principle Component Analysis (PCA) was performed on 129 landmarks duly registered using Procrustes analysis and only 11 Principle Components (PCs) were retained. Using DFA, the authors classified facial sex with an accuracy of 96.8% in the first dataset and 98.7% in the second dataset. Discriminant function scores were used as the masculinity index. The relationship between these objective scores and perceived attractiveness was then analysed. The authors did not find any correlation between the male facial masculinity index and perceived attractiveness. However, the relationship between masculinity and attractiveness in female faces was significant and negative. Using the same approach, Stephen et al. [22] measured the masculinity index of 34 male participants using their 2D images. Interestingly, the authors found no correlation between their objective measure of sexual dimorphism and perceived attractiveness. Perhaps the absence of correlation is due to the fact that the authors have used 2D texture images in their experiments. Distances on 2D images are unable to model the facial surface accurately.

The above mentioned studies, on the one hand, highlight the importance of gender rating in evaluating various psychological and medical aspects in humans, and on the other hand, present the obvious difficulty in obtaining these scores. Our literature review shows that, so far, the methods employed for measuring objective masculinity/femininity scores fail to explain the underlying processes in perceptual gender scoring. That is why the objective scores obtained using these methods do not correlate well with subjective perceptual scores, making it difficult to use them instead of, or in combination with, perceptual scores in different studies. Note that, the main aim of these studies was to find relationship between different characteristics/attributes of the face with perceived (or objective) facial gender scores instead of looking for a direct relationship between their perceptual and objective facial masculinity/femininity. The requirement, therefore, is to understand the facial features used by humans to score the masculinity/femininity from faces and to evaluate the plausibility of reproducing these scores using objective measures. Once reliable objective measures are established, computer algorithms can be used to predict the perceived masculinity/femininity of a face with high confidence.

Understanding human perception or Human Visual System (HVS) for particular tasks has been of great interest to researchers (Note that, “Human Visual System” also refers to the anatomical structure of the visual system. However, throughout this paper we have used this term to refer to the cognitive mechanism employed by the human mind to perceptually asses and analyse visual information). Bruce et al. [23] performed Discriminant Function Analysis (DFA) for sex classification using 2D and 3D Euclidean distances extracted from 73 landmarks, the ratios of these distances and angles between them. The authors suggested that perhaps the human visual system takes into account a subset of 16 measurements to classify facial sex, since these features result in a classification accuracy of 94%. Similarly, to understand human and machine sex classification behaviour, Graf et al. [24] used 2D images as stimuli to perform perceptual as well as computational sex classification. The authors asked human subjects to visually classify the 2D images for sex. Next, they used the Principle Components of the images and several state of the art classifiers to understand human internal decision space for sex classification.

To the best of our knowledge, there is no exclusive work on understanding the broad features used by HVS to give a measure to the degree of masculinity/femininity of the face. In the absence of such an understanding, the objective scores calculated by researchers, as evident from our survey, either do not correlate significantly with the perceptual scores or go against the established findings on relationship between perceived sexual dimorphism and other facial traits. This research gap has also resulted in the lack of development of robust algorithms for objective scoring of masculinity/femininity.

There are two major cues used by humans for facial sex classification: shape and appearance. Given the 3D nature of the face, a large amount of shape information gets lost in the 2D images of the face. On the contrary, a 3D face image, although more difficult to capture, has more shape-rich information. O'Toole et al. [25] showed that 3D geometric information outperforms the texture in classifying sex of a face. Similarly, Bruce et al. [26] claimed that visually-derived semantic information like age, expression, gender etc. depend mainly on the geometric form of the perceived face. Therefore, we focus on using 3D geometric faces in this work to capture human perceptual ratings on gender. The main research questions that we want to address are the following:

- Which geometric features are used by the HVS in perceiving the degree of gender of a 3D face?
- Can a mathematical model mimic human performance and objectively rate the gender of a 3D face?

The answers to these questions will help in understanding facial sexual dimorphism and the diagnosis of related syndromes. In this study, we present 3D face models of 64 subjects in frontal, oblique and profile views to 75 raters to obtain perceptual ratings and analyse the physical features used by the raters to rate the faces. Next, we build a computational model based on the results of the perceptual study to objectively rate the gender using 3D Euclidean and geodesic features and their combinations. Using this model, we present our findings on the nature of geometric features used by the HVS in rating gender. Our results suggest that humans take into account a combination of 3D Euclidean and geodesic distances while perceiving the amount of sexual dimorphism in a face.

## Materials and Methods

This study was performed at University of Western Australia (UWA) and Princess Margaret Hospital (PMH). All participants completed an informed consent form having been given written and verbal details of the tasks to be completed. The study was approved by the Princess Margaret Hospital Ethics Committee vide Approval Reference Number: 1532/EP. For developing the mathematical model for objective gender scores, the digital data was analysed anonymously. All identification features like the meta-data, texture etc. were stripped from the 3D images before hand.

### Subjects

Images were obtained from participants recruited from the student body of UWA. 3D images of a total of 64 participants between the ages of 18 and 25, of varying population affinities, who had not undergone significant craniofacial surgery, and had no craniofacial abnormalities or injuries were captured for the current study. The self-reported population affinities were grouped into two categories of Europeans (Caucasian) and non-Europeans (‘Other’).

Fifty two percent of 64 subjects were females and 48% were males. 80% of the faces were Caucasian/European. The remaining 20% were allocated to the ethnicity category “other” which included Asians (n = 6), Blacks(n = 1), Anglo-Indian (n = 1), Eurasian (n = 2) and Indo-Chinese (n = 1). The majority (78%) of rated faces were of people between the ages of 18 and 21. Sixty eight percent of the rated subjects were born in Australia. Fourteen percent of these identified themselves as having an ethnicity other than Caucasian. The majority of the “other” group were born in Australia (46%), or in Asia (38%), the remainder having been born in Africa (n = 2). Caucasians born outside of Australia were born in Africa (n = 2), New Zealand (n = 6), and the UK (n = 6).

### Raters

Raters of a similar background to the imaged subjects were recruited from within and outside the student body at The University of Western Australia. These raters were also categorised as European/Caucasian or non-European/Other.

The panel of raters (n = 75) was composed of 40 females (53%) and 35 males (47%). Sixty four of the raters were Caucasian/European (84%). The majority, n = 48 (64%), of raters were aged between twenty one and twenty three, although the full age range extended from eighteen to twenty five. The mean age of the raters (21.9 years) was greater than that of the rated image subjects (19.9 years) 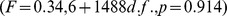. Seventy seven percent of all raters were born in Australia. Seven percent of these identified themselves as having an ethnicity other than Caucasian/European. The majority of the ethnic group Other/non-European was born in Asia (58%), or in Australia (33%), the remainder having been born in Africa (n = 1). Europeans born outside of Australia were born in Asia (n = 2), New Zealand (n = 2), and the UK (n = 4).

### 3D Facial Stereophotogrammetry

Three dimensional (3D) images of the faces of participants were captured using the 3dMDface 3D stereophotogrammetry system (3dMD LCC, Atlanta Georgia, USA). The 3dMDface system generates 180 degree (ear to ear) 3D images by employing the technique of triangulation. These high-resolution images are captured within 1.5 milliseconds (ms) [27]. Image capture was undertaken in an office environment under standard clinic/office lighting conditions. Subjects were positioned so that imaging of the full face from ear to ear could be achieved. Images were taken of participants with faces holding a neutral expression, and jaws in centric relation with temporomandibular joint seated and natural dental contact without clenching force.

### Stimuli Preparation for Perceptual Scoring

Texture maps were stripped from the 3D images to remove features such as eyebrow shape and skin colour. Facial surface was smoothed to diminish the effects of skin texture and eyebrow coarseness. This is done in order to ensure that the raters' perceptions are based solely on facial geometry.

Processed images were prepared into individual packages of 20 randomly chosen faces for viewing on a visual display unit by each individual rater. Packages comprised equal number of males and females, drawn randomly from sex and population subgroups.

### Stimuli Preparation for Objective Scoring

**Figure 2:**
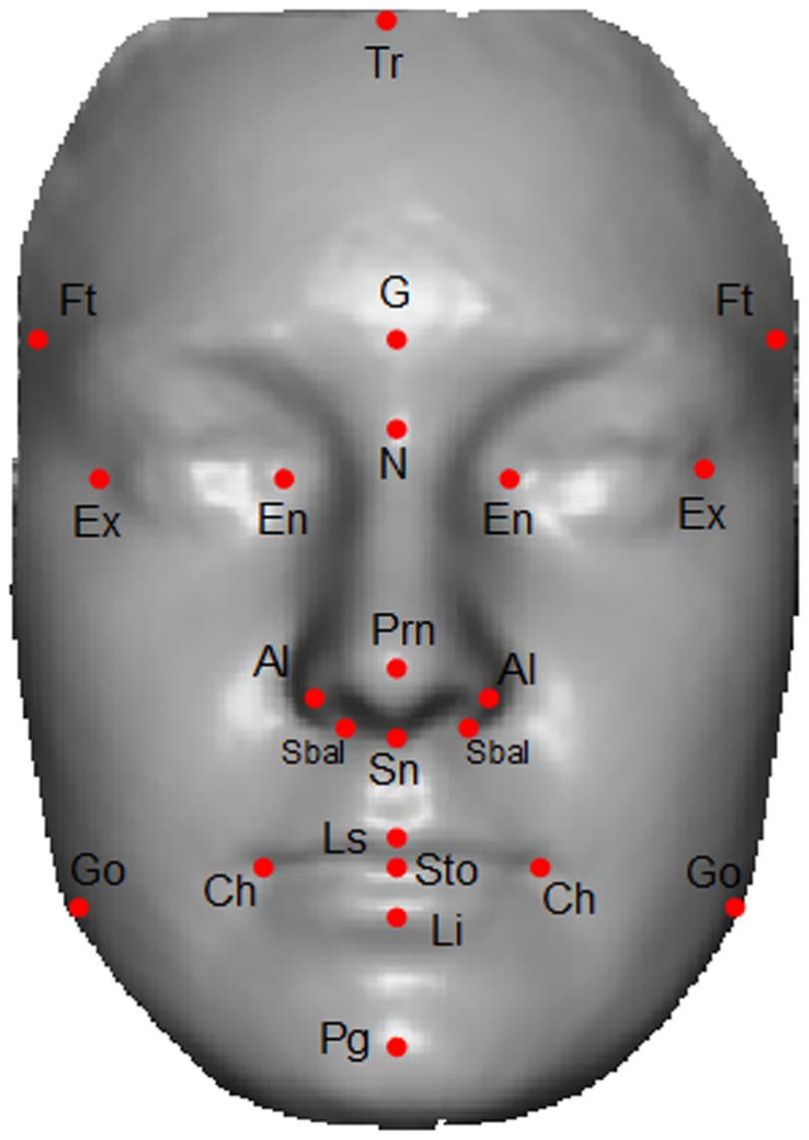
Landmarks used in our algorithm. 23 landmarks annotated on a shaded textureless 3D image. The image is the average face of 10 male subjects from our database.

We annotated 23 biologically significant landmarks [28] on each image as shown in Figure 2. The motivation for using these landmarks comes from the fact that they represent the sexual dimorphism of the face [29]. These landmarks and Euclidean distances measured from them are used to measure a quantitative dimension for the morphological deviation from the normal face [28], to delineate syndromes [30] and to measure objective masculinity/femininity [21]. We have selected the facial landmarks that relate to the bony structure of the face which is effected by the ratio of testosterone to estrogen (oestrogen) during adolescence [31]. It is believed that facial masculinity is associated with levels of circulating testosterone in men [19]. Hence it is intuitive to use features extracted from these bony landmarks for facial gender scoring.

**Figure 3:**
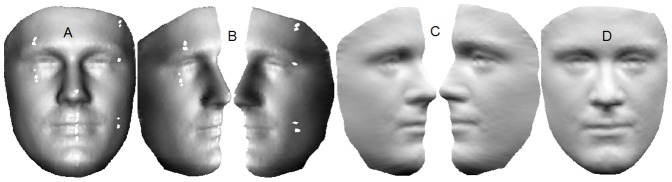
Different steps in preprocessing. (A) The raw input face. (B) Bisected raw face rotated by 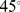. Notice the holes in the eye region. (C) Processed face. (D) Processed face stitched back seamlessly.

The pose of each 3D face is corrected to a canonical form based on four landmarks (Ex(L), Ex(R), N and Prn). This step is required to eliminate any error due to pose in the extraction of geodesic distances which will be discussed in detail in the Study 2 of the Experiments Section. Holes are filled and noise removed by re-sampling the 3D face on a uniform grid using the gridfit [32] algorithm. Since some portions of the face are expected to be self occluded (e.g. region around Ac) when re-sampled on a grid, we bisect the 3D face along the vertical axis at the nose tip and rotate each half by 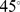 before re-sampling to mitigate this problem. Besides hole filling, another advantage of bisecting and rotating the halves before re-sampling is that the resulting 3D face has a more uniform sampling in the 3D space. The processed halves are then rotated back and stitched seamlessly to form a single mesh. Figure 3 shows the different preprocessing steps.

### Evaluation Criteria

The main focus of this paper is to find geometric features that are used by HVS for rating gender. Since it is well known that texture itself is very informative on sex classification [20], we used textureless 3D rendered images to avoid any bias in the results due to texture. Abdi et al. [33] show that hair is one of the major contributors in sex classification. To avoid bias resulting from this feature, ratings were obtained on 3D images with the hair concealed or cropped.

Consequent to the above considerations, raters were asked to rate each of the 64 faces for perceived masculinity/femininity and nominate the facial regions they used for this judgement. A computational model was then developed based on this study to objectively score the gender. Our evaluation criterion is the correlation between perceptual ratings and objective scores from the model. Given two random variables 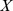 and 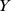 with 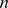 samples each, their correlation 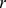 is defined as,
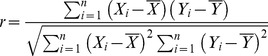
(1)


In each study, we depict the correlation for males and females in a plot. We also project the objective and subjective perceptual scores on a Bland-Altman plot [34]. Bland et al. [34] proposed a technique for comparing the outcome of two methods in clinical practice. They argue that a comparison between the average of the outcomes to the difference is a better way of assessing the agreement between two different methods. The Cartesian coordinates of the Bland-Altman plot 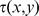 are given by,
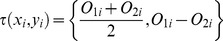
(2)where 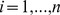 are the samples of each observation 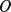 belonging to male/female class.

### Perceptual Scoring

**Figure 4:**
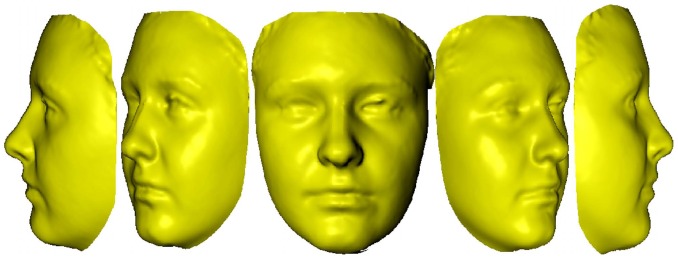
Facial views for perceptual rating. Series of facial views of each subject shown to raters. From left to right: left profile, left oblique, straight, right oblique, right profile.

As mentioned earlier, the stimuli were prepared into individual packages of 20 randomly chosen faces for viewing on a visual display unit by each individual rater. The rater was unaware of the sex and population composition of the package. As shown in Figure 4, a series of five facial views: left profile, left oblique, straight, right oblique, right profile, were prepared for each subject and displayed on the screen. Raters were able to toggle between these images in making their ratings.

Questionnaires were presented and filled out electronically while viewing the images on a second computer screen. Raters were asked to do the following

- Fill out a personal information questionnaire detailing age, sex and population affinity.
- View each face and rate the degree of masculinity or femininity of the face on a 20 point scale.
- Nominate the facial regions that they used to make their judgement. The options available were forehead, eyes, nose, cheeks, mouth, chin, jaw and no specific features.
- Identify the sex of the individual depicted.

### Objective Scoring

**Figure 5:**
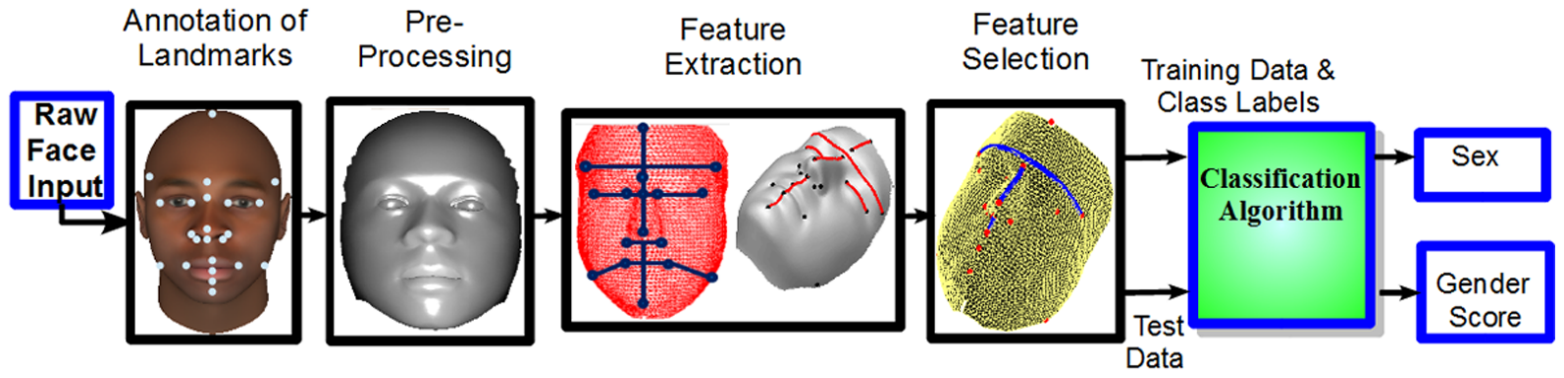
Block Diagram. Block diagram of the proposed gender classification and scoring algorithm. For details see the Objective Scoring Section. The synthetic images are from [45], [46].

**Figure 6:**
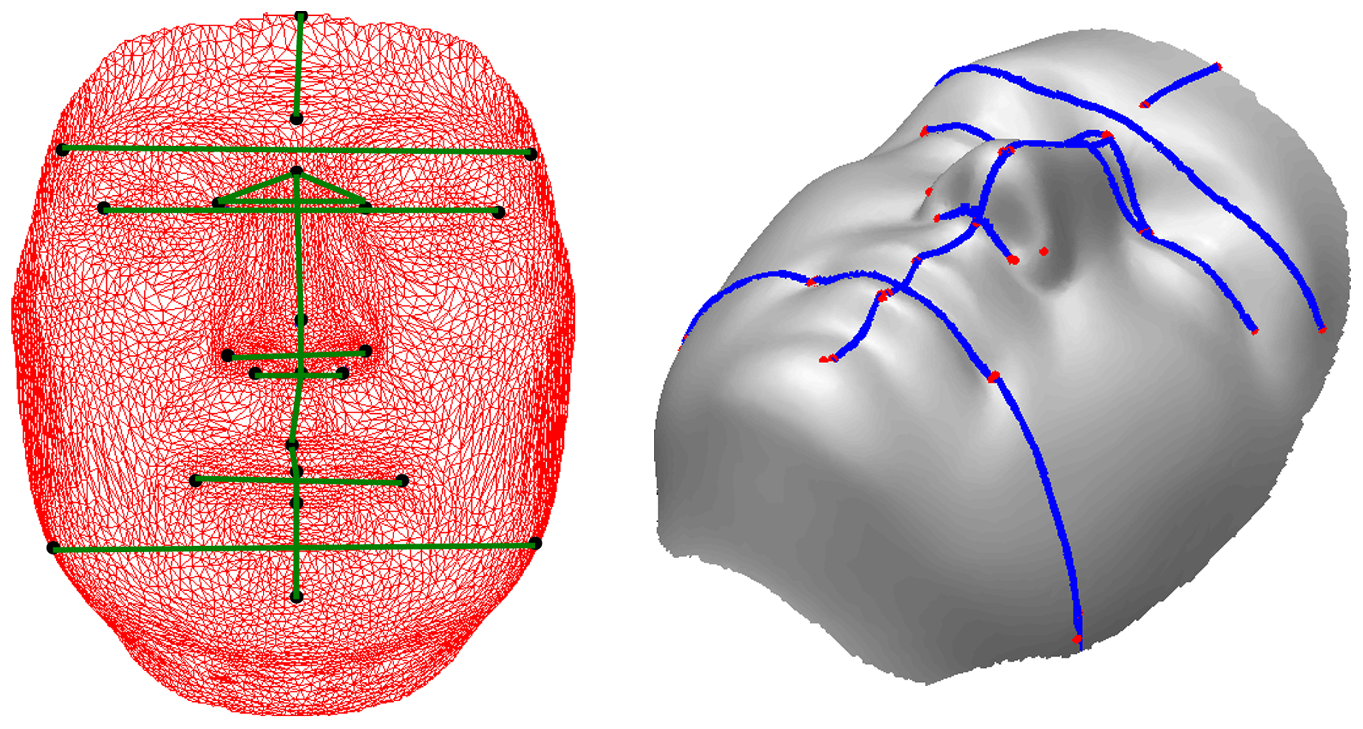
Features used in our algorithm. Some of the 3D Euclidean (left) and geodesic (right) distances used in gender scoring algorithm.

An overview of our gender scoring algorithm is given in Figure 5. Gender classification is an important prerequisite for obtaining objective gender scores. Using the annotated landmarks, 44 distances (22 each of the 3D Euclidean and geodesic) related to the regions indicated in Table 2 were extracted as features. Figure 6 shows some of the features used. Further details on these features are given in the Experiments Section.

**Table 2:** Significant facial features in perceptual gender scoring.

Feature	*x* ^2^	p	Masculinity/Femininity association
**Forehead**	5.28	0.071	No particular association
**Eyes**	23.69	<0.001	Femininity
**Nose**	3.08	0.214	No particular association
**Cheeks**	36.39	<0.001	Femininity
**Mouth**	23.63	<0.001	Femininity
**Chin**	19.38	<0.001	Masculinity
**Jaw**	58.29	<0.001	Masculinity
**No Spec**	2.97	0.227	No particular association

Chi-square and propability values for the correlation between facial features and their use in rating masculinity/femininity.

We begin with feature selection which is a process of selecting the most relevant features for classification while removing the redundant ones. For this purpose we use the minimal redundancy maximal relevance (mRMR) algorithm packed in a forward-selection wrapper [35]. The algorithm first calculates the intrinsic information (relevance) within a feature and also the mutual information (redundancy) among the features to segregate different classes. Then it maximizes the relevance and minimizes the redundancy simultaneously. Let 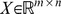 be the feature matrix with 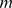 observations and 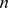 features, 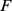 be the target reduced feature set and 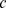 be any arbitrary class from the set of classes 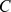, then relevance is defined by,
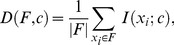
(3)and redundancy is defined by,
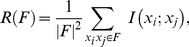
(4)where 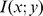 is the mutual information between 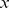 and 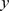. Maximal relevance and minimal redundancy is obtained by taking the maximum and minimum values of (3) and (4) respectively. The goal of simultaneously maximizing the relevance and minimising the redundancy is achieved by maximizing the function 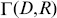 where,
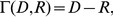
(5)or
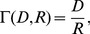
(6)where equation (5) is the Mutual Information Difference and equation (6) is the Mutual Information Quotient formulation of mRMR algorithm. Since our feature set is small, we find the classification accuracy yielded by both formulations and use only the one giving the maximum accuracy on training data. The reduced number of candidate features 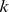 is selected by first obtaining 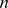 feature sets 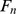 using the mRMR sequential search (Eq. 5 or 6 depending on which one gives better accuracy). More specifically 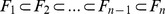. Next we compare the classification accuracy for all feature subsets 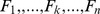 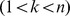 to find a range for 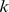 where the classification accuracy is maximum. Finally, we select a compact set of features by exploiting the forward-selection wrapper [36]. The wrapper first searches for a single feature 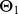 from the feature set 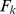 which gives the maximum classification accuracy. Then, from the subset 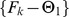 we search for another feature such that the subset 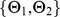 gives the maximum accuracy irrespective of the previous one. This is a deviation from the original mRMR algorithm [35] which desires a feature subset that produces better or equal accuracy than the previous subset in order to minimize the number of evaluations due to the greater number of candidate features in 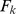. Since our original feature set 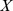 contains fewer than 50 features and the size of candidate feature set 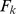 is even smaller than 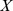, therefore, we let the wrapper evaluate all possible subsets of 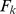 in a forward selection scheme enabling us to find the reduced feature subset that gives the best accuracy. Consequently, we obtain a feature set 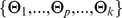 where 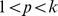 and we select the feature subset 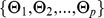 which corresponds to the highest accuracy. Note that this is the most compact feature subset as 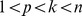.

We train a Linear Discriminant Analysis (LDA) classifier using an exclusive set of training data. Let 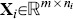 be the matrix of features of class 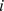 with 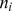 samples. LDA maximizes the ratio of *between-class scatter* to *within-class scatter*. Between-class scatter is defined as
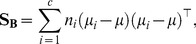
(7)and within-class scatter is defined as
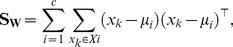
(8)where 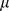 is the mean of all classes, 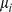 is the mean of class 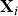 and 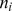 is the number of samples in 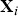. Fisher [37] proposed to maximise the ratio between 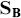 and 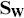 relative to the projection direction by solving
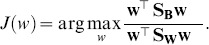
(9)


By differentiating the equation with respect to 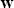 and equating it to zero, we get 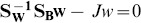, which is a generalized eigenvalue problem and the eigenvector 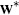 of 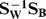 is the desired optimal direction. Given the learnt LDA projection 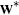, a query face is classified into one of the two genders. The projection of feature vector 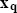 (of a face with unknown gender) on the LDA space is given by 
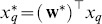
.

Gender classification is performed based on the distance between the 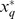 and the means of the projected classes 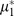 and 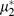 such that
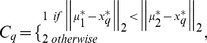
(10)where 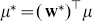


**Figure 7:**
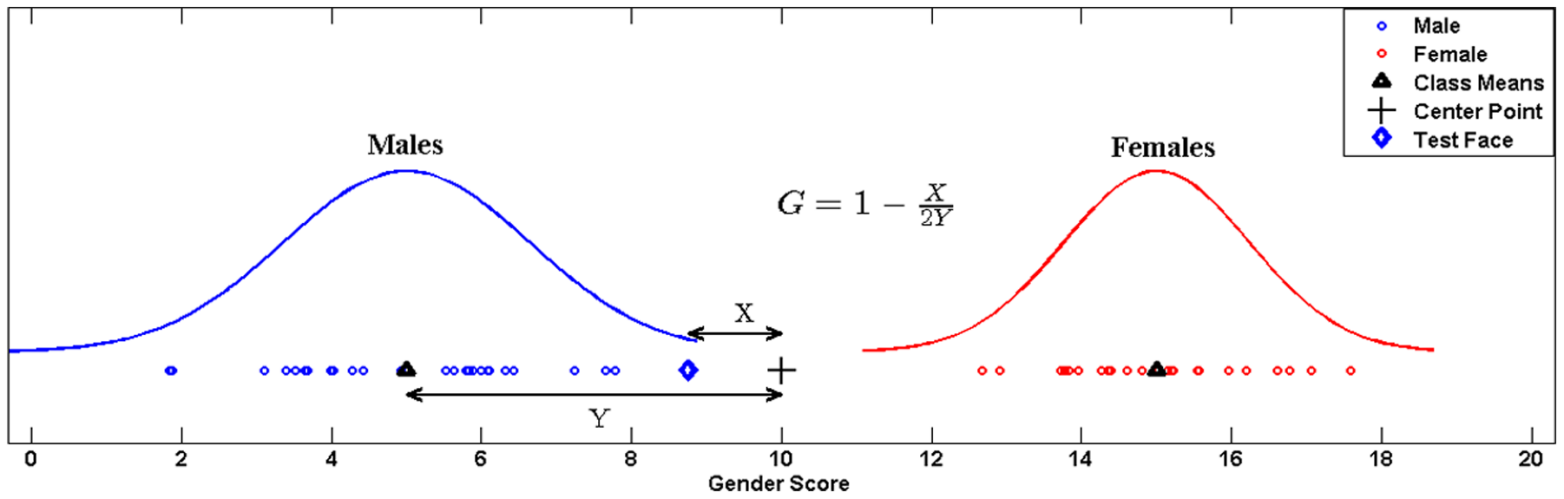
Gender scoring in LDA projected space. Diagram depicting the process of objectively scoring the gender in LDA space to assign a metric for masculinity/femininity of the test face.

Interestingly, the directional distance of a projected test face from the center of the projected means of the two classes gives an intuitive insight into the amount of masculinity or femininity of the face. Let 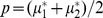 be the center of the projected means.The gender score 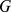 of a test face 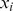, whose gender has already been determined with Eqn. 10, is defined as
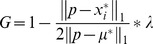
(11)where 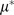 is the projected mean of either class (1 or 2) and 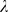 is a scaling factor for comparability with the available human perceptual ratings. In our case 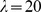. Hence we score the gender on a scale of 0 to 20 (0 being most masculine and 20 being most feminine). Figure 7 illustrates the process of scoring the gender of a query face in the LDA projected space.

## Results and Analysis

### Perceptual Scoring

**Figure 8:**
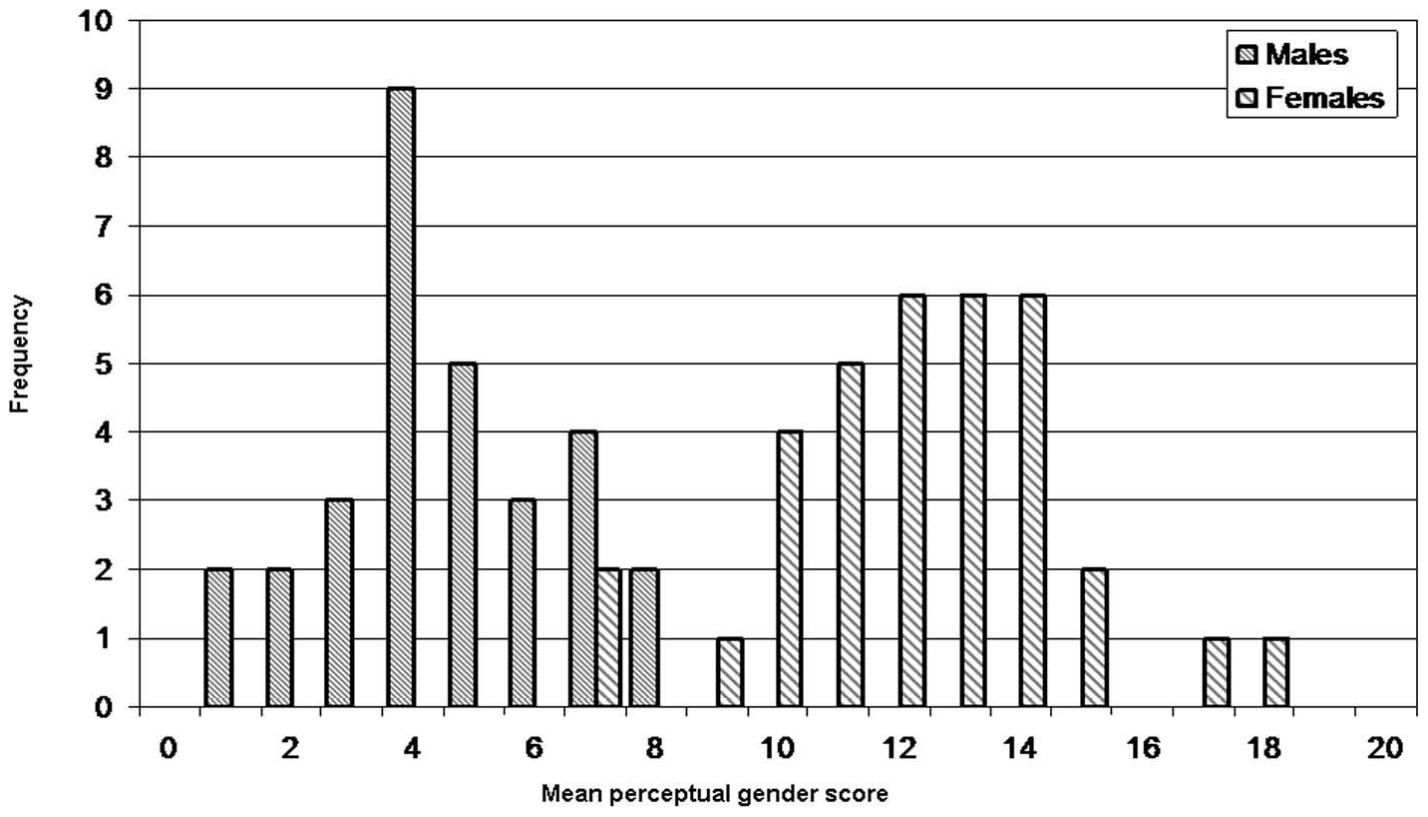
The perceptual subjective gender scores. A histogram of mean perceptual masculinity and femininity ratings obtained from 75 raters.

While ratings of masculinity/femininity were clearly bimodal (Figure 8) with most males rated at the lower one third of the scale, and most females in the upper one third, a substantial proportion of images (29%) were rated in the middle one third, or perceived to be ambiguously masculine/feminine. The ratings from all the 75 raters were found to be significantly consistent (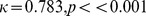) using the Fleiss Agreement Test [38].

The sex and ethnicity of the person represented in images had a significant influence on how they were rated by all groups 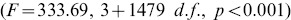. In general the perceived masculinity or femininity of the imaged subject was independent of the background of the person doing the rating. Both European male and female faces were considered to be more masculine than their non-European counterparts.

There was a strong tendency for the chin and jaw to be nominated as significant indicators in judgements of faces rated as extremely masculine (ratings 0 to 4), while the eyes, cheeks and mouth were the most frequently nominated features used in judgements of faces receiving high femininity ratings (ratings 15–20). Table 2 gives the detailed test values for each feature.

Gender was correctly identified in 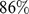 of the instances. All sex and ethnic groups had the same ability to identify gender overall 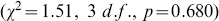. Raters were adept at correctly identifying sex for their own ethnic group (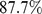 correct). Raters were slightly better at identifying the sex of the dominant culture when they were a minority born amongst the dominants than if they were a member of the dominant culture trying to identify the sex of one of the minorities (Europeans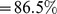 correct; non-Europeans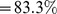). Europeans were better at classifying the sex of non-Europeans (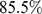 correct) than non-Europeans were at classifying the sex of Europeans (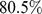) 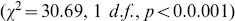. Gender identification errors were more likely to be made amongst female faces (23% wrong) than amongst male faces (10% wrong) 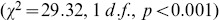. In particular, there was a strong tendency for female Europeans to be wrongly identified as males (29% wrong), while male Europeans (5% wrong) were very unlikely to be mistaken for females 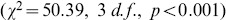. Correctly identified females were perceived to be significantly more feminine than those that were mistaken for males 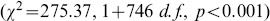. Correctly identified males were perceived as more masculine than those mistaken for females 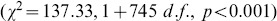. The ability to identify sex did not improve with the number of faces that were viewed 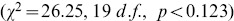.

### Objective Scoring

#### Study 1: Euclidean Measurements.

Our first study constitutes obtaining objective gender scores using 3D Euclidean distances. Let 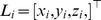 be the 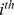 landmark. The 3D Euclidean distance 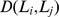 between landmarks 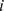 and 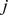 is defined as,
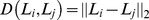
(12)


Figure 6(Left) shows some of the 3D Euclidean distances used in this experiment.

**Figure 9:**
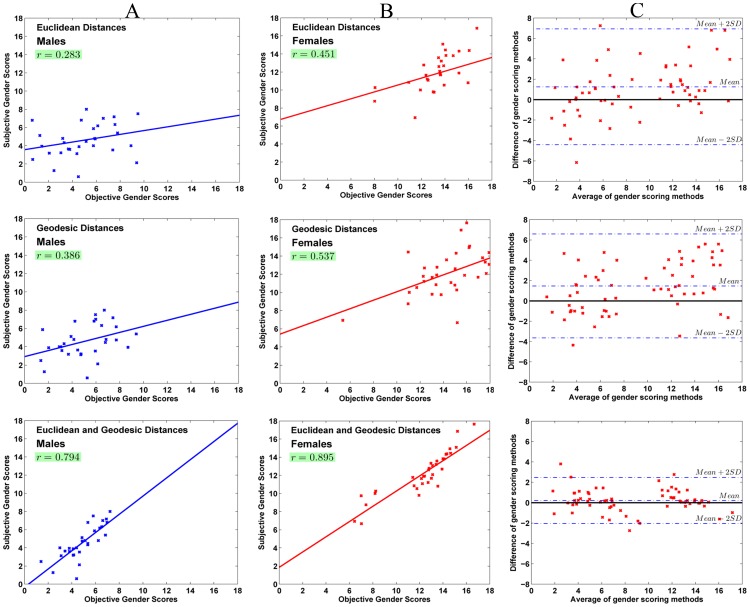
Results of objective gender scoring. (A) Correlation for males. (B) Correlation for females. (C) Cumulative Bland-Altman plot. Correlation and Bland-Altman plots between objective and subjective gender scores for males and females using only 3D Euclidean distances (First Row), only geodesic distances (second row) and combination of Euclidean and geodesic distances (third row).

Using 3D Euclidean distances as features, our proposed algorithm classifies 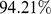 subjects correctly as males or females. The correlation between objective gender scores and the perceptual scores is 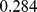 and 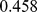 for males and females respectively. Figure 9(a & b, first row) show the correlation and best fit line for males and females while Figure 9(c, first row) shows the Bland-Altman plot between the objective and perceptual subjective scores.

It is evident that objective scores for masculinity and femininity do not correlate well with the perceptual subjective scores. In Figure 9(c, first row) ideally the mean of the difference of objective and subjective gender scores should have been zero. However, we can see that the mean difference line is well above zero and the width of the limits of agreement in this case is 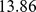.

**Figure 10:**
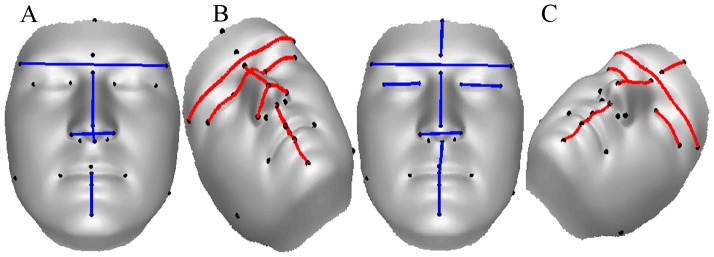
Most discriminating features between males and females found in the three experiments. (A) Euclidean distances only. (B) Geodesic distances only. (C) Combined Euclidean and geodesic distances.

Clearly, 3D Euclidean distances do not seem to be the features that HVS concentrates on while scoring the facial gender. However, it is interesting to note that the forehead width (Ft-Ft), nasal bridge length (N-Prn), nasal tip protrusion (Sn-Prn), nasal width (Al-Al) and chin height (Sto-Pg) are selected as the most differentiating features by our algorithm (see Figure 10(a)). This is in line with the findings of Burton et al. [20] who performed experiments on a subset of 2D and 3D Euclidean distances. Note that the authors handpicked these features based on knowledge from existing literature, whereas our approach relies on a mathematical feature selection algorithm. This endorses the mathematical model we use for obtaining discriminant features.

#### Study 2: Geodesic Measurements.

**Figure 11:**
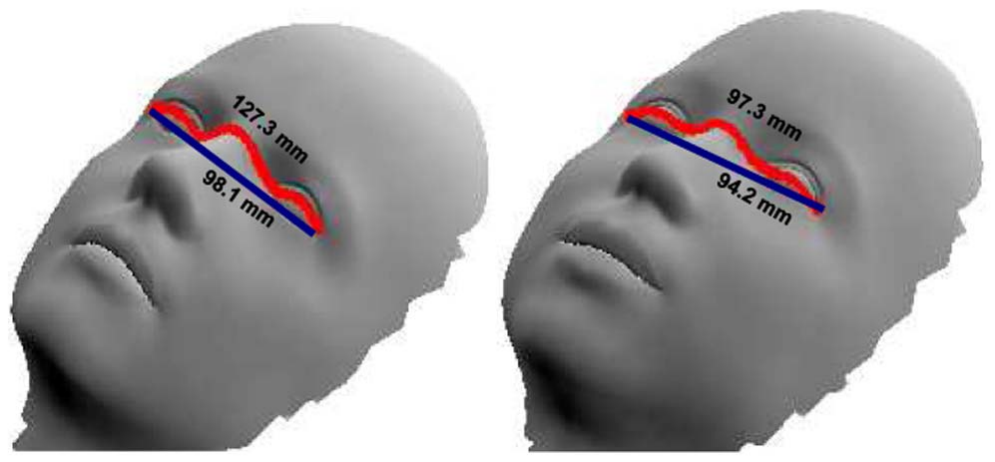
Robustness of geodesic distances to facial expression. Geodesic and 3D Euclidean distances of biocular width shown on two models. Left model has a protuberant nose and hence a greater geodesic distance than the right model which has a flatter nose. Euclidean distance in both the models is similar.

**Figure 12:**
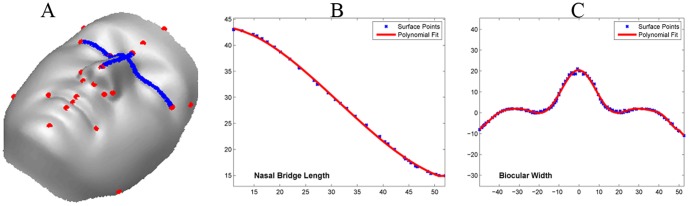
Modelling of geodesic curves. (A) Geodesic curves for nasal bridge length(N-Prn) and biocular width(Ex-Ex). (B–C) Fitting polynomials to these curves. Notice that N-Prn is modelled by a fourth order curve while Ex-Ex is modelled by a 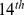 order curve.

In the second study, we use geodesic distances to predict the facial gender scores. Some examples of the geodesics can be seen in Figure 6(Right). We define geodesic distance 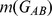 between points 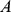 and 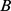 as the length of the curve 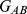 generated by orthogonal projection of the Euclidean line 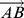 on the 3D facial surface. This is precisely the reason for normalising the pose of each 3D face as variation in pose can present a different surface to the viewing angle. Less curved distances like the upper lip height (Sn-Sto) are modelled by a second order polynomial while more curved distances with multiple inflection points, like the biocular width (Ex-Ex) are modelled by higher order polynomials. Studies suggest that geodesic distances may represent 3D models in a better way as compared to 3D Euclidean distances [39]. Gupta et al. [40] argue that algorithms based on geodesic distances are likely to be robust to changes in facial expressions. In support of this argument Bronstein et al.[41] have suggested that facial expressions can be modelled as isometric deformations of the 3D surface where intrinsic properties of the surface like geodesic distances are preserved. Figure 11 depicts the variation in 3D Euclidean and geodesic distances in biocular width on two models. The left model has a protuberant nose and hence a larger geodesic distance than the right model which has a flatter nose. Euclidean distance in both the models is similar. Figure 12(a) shows some of the extracted geodesic features and Figure 12(b–c) show the process of fitting a polynomial to these features.

Geodesic distances classify facial sex with an accuracy of 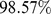. The correlation between objective gender scores and the perceptual subjective scores also increases to 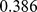 and 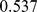 for males and females respectively. Figure 9(a & b, second row) show the correlation and best fit line for males and females while Figure 9(c, second row) shows the Bland-Altman plot between the objective and perceptual subjective scores.

Even though the correlation has improved, the geodesic distances alone do not seem to be the features of choice for HVS while ascribing a score to facial gender. Figure 9(c, second row) shows that the mean of the difference is still well above zero and the width of limits of agreement in this case is 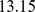. Once again the forehead width (Ft-Ft), nasal bridge length (N-Prn), nasal width (Al-Prn-Al) and chin height (Sto-Pg) are amongst the most differentiating features. However, with geodesic distances, the upper lip height (Sn-Sto), eye fissure length (Ex-En) and intracanthal width (En-En) are added as the most discriminating sex classification features (see Figure 10(b)).

#### Study 3: Combined Measurements.

In the last experiment, we use a combination of 3D Euclidean and geodesic distances as our features for gender scoring. Since most of the gender discriminating features are common between the two families of distances, it seems intuitive to combine them and analyse their effect.

Equipped with a combination of 3D Euclidean and geodesic distances, our algorithm classifies facial sex with an accuracy of 99.93%. There is also a significant boost in the correlation between the objective and subjective gender scores which now is 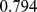 and 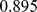 for males and females respectively. The Bland-Altman plot shows the mean of the difference between the two scoring methods to be 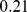 while the width of limits of agreement is 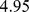. This is a significant improvement as compared to the previous experiments. Figure 9(a & b, third row) show the correlation and best fit line for males and females while Figure 9(c, third row) shows the Bland-Altman plot between the objective and perceptual subjective scores.

The most differentiating features between the two sexes are once again common between the two families of distances. The Euclidean and geodesic distances for forehead width (Ft-Ft), nasal bridge length (N-Prn), nasal width (Al-Al), eye fissure length (Ex-En), chin height (Sto-Pg) and upper lip height (Sn-Sto) are the most discriminating features in our algorithm (see Figure 10(c)). However, this time the forehead height (Tr-G) is added to the list of discriminating features.

The above results suggest that the human visual system looks at the combination of Euclidean and geodesic distances between certain features on the face to give a gender score.

## General Discussion

In the three studies involving various families of features, we have tried to find the predictors that the human visual system uses to attribute a measure to the facial gender. Beginning with 3D Euclidean distances alone, we see that there is little correlation between objective gender scores and subjective scores. This situation improves slightly when geodesic distances are used. The reason is straight forward as geodesic distances can model the facial surface curvature better than the Euclidean distances. However, the results are still below an acceptable significance threshold. Finally, when we use a combination of Euclidean and geodesic distances we see that the correlation between the two methods of scoring improves significantly and so does the agreement between them. This seems to corroborate the claim of Bruce et al. [23] that humans use a combination of predictors to perceive the sex of a face. Furthermore, as is evident from Figure 10(c), the most discriminating features from both families of distances seem to be common. This indicates that HVS might actually be taking into consideration the ratio between 3D Euclidean and geodesic distances while making a decision on the gender score of a face.

Relating the sex classification results to the gender scores in the three studies gives us a very interesting clue. In all three studies, sex classification results are very impressive. In fact, the base accuracy of 94.21% using only the 3D Euclidean distances tends to agree with the findings of Burton et al.[20] who classified facial sex with 94% accuracy using 2D and 3D Euclidean distances. However, the objective gender scores obtained with this family of distances do not significantly agree with the perceptual scores. Even when the classification results improve to 98.57% using the geodesics, the correlation between the objective and subjective gender scores remains below an acceptable significance threshold. This trend changes significantly when a combination of the two families of distances is used as predictors even though the sex classification results improve by 1.36% only. It shows that even though facial sex can be classified accurately using only the 3D Euclidean or geodesic distances, perfect and more meaningful gender scores can only be obtained when a combination or ratio of these distances are taken as features for gender scoring.

Commenting on the method of obtaining gender scores, it is observed that a classification algorithm is a necessary prerequisite. However, the scoring result itself is invariant to the sex classification accuracy. This is evident from the gender scores obtained for females in the three experiments. There are a few female subjects who score below the boundary line of 10 giving them a more masculine gender score. This is indicative of a failure in classifying their sex but correlates very well with the perceptual subjective scores. Therefore, even though the algorithm misclassifies their sex, it still gives them a meaningful gender score which tends to agree with the subjective scores. Hence, our proposed algorithm puts the facial gender in the category of a continuum rather than binary.

From the Categorical Perception (CP) point of view, our results corroborate the findings of Armann and Bülthoff [42], that there is no evidence for naturally occurring CP for the sex of faces. Results of perceptual scoring, although bimodal, show that the gender ratings are on a continuum and do not follow a decision boundary. Consequently, a few female subjects were rated more masculine, hence crossing the decision boundary. This trend was replicated by our proposed computational model which ascribes the correct gender scores to even those subjects which fall on the other side of the decision boundary. Furthermore, the participants in Armann and Bülthoff's study [42] show a consistent bias to judge faces as male rather than female. Our findings from perceptual sex classification replicated this observation as we found a strong tendency for female Europeans to be wrongly identified as males (29% wrong), while male Europeans (5% wrong) were very unlikely to be mistaken for females 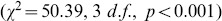.

Our choice of features was motivated by the results from perceptual scoring. Instead of taking 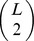 combinations of distances, where 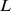 is the number of landmarks, we developed our model around the facial features that our raters indicated were instrumental in giving a score. It is evident from Figure 10(c) that our algorithm also selects the features that were significant in subjective perceptual scoring. However, distances relating to the jaw (Go-Go) and mouth (Ch-Ch) were not highly discriminating. While there is no plausible reason for the mouth width (Ch-Ch) to be excluded from the list, mandible width (Go-Go) may have been excluded due to localization error of the related landmarks. Gonions (Go,L and Go,R) are a palpable landmarks indicating the extremes of the jaw and as such are very difficult to annotate consistently on 3D images.

Facial rating for attractiveness and sexual dimorphism plays an important role in planning reconstructive and cosmetic surgery. This procedure depends on a number of physiological and psychological constraints, like, age, sex, health state, structure, shape of the face and patient's needs and expectations. Patients who undergo such procedure are rated by human observers pre and post surgery to assess any improvement in perceptual attractiveness [14], [15]. With the development of 3D simulation techniques to preview the aesthetical results of facial cosmetic surgery [43], our proposed algorithm can assist in predicting the attractiveness of the surgical outcome as it correlates significantly with human perceptual results. For example, secondary rhinoplasty is a nose operation carried out to correct or revise an unsatisfactory outcome from a previous rhinoplasty [44]. Lee et al. [8] have proposed a three-dimensional (3D) surgical simulation system, which can assist surgeons in planning rhinoplasty procedures. Our proposed algorithm can be used in such cases to assess the improvement in facial attractiveness of the resulting rhinoplasty through gender scoring, thus reducing the chances of further secondary procedures.

We can conclude by claiming that our proposed algorithm helps us in a better understanding of the Human Visual System. It is the first algorithm that has such a significantly high correlation with the mean perceptual scores given by 75 raters on 64 subjects. Hence, it may be possible to use these gender scores in a myriad of applications in medical and psychological fields where human raters are employed to obtain these scores.
